# A Novel Orthohepadnavirus Identified in a Dead Maxwell’s Duiker (*Philantomba maxwellii*) in Taï National Park, Côte d’Ivoire

**DOI:** 10.3390/v11030279

**Published:** 2019-03-19

**Authors:** Jan F. Gogarten, Markus Ulrich, Nishit Bhuva, Joel Garcia, Komal Jain, Bohyun Lee, Therese Löhrich, Alexandra Oleynik, Emmanuel Couacy-Hymann, Terence Fuh Neba, Nischay Mishra, Thomas Briese, Sébastien Calvignac-Spencer, W. Ian Lipkin, Fabian H. Leendertz

**Affiliations:** 1 Epidemiology of Highly Pathogenic Microorganisms, Robert Koch Institute, 13353 Berlin, Germany; jan.gogarten@gmail.com (J.F.G.); UlrichM@rki.de (M.U.); tloehrich@wwfcar.org (T.L.); 2 Viral Evolution, Robert Koch Institute, 13353 Berlin, Germany; 3 Center for Infection and Immunity, Mailman School of Public Health, Columbia University, New York, NY 10027, USA; npb2123@cumc.columbia.edu (N.B.); jg2876@cumc.columbia.edu (J.G.); kj2230@cumc.columbia.edu (K.J.); bl2493@cumc.columbia.edu (B.L.); ap2811@cumc.columbia.edu (A.O.); nm2641@cumc.columbia.edu (N.M.); 4 Institute of Microbiology and Epizootics, Freie Universität Berlin, 14163 Berlin, Germany; 5 World Wide Fund for Nature, Dzanga-Sangha Protected Areas, Bangui BP 1053, Central African Republic; TNeba@wwfcar.org; 6 Laboratoire National D’appui au Développement Agricole/Laboratoire Central de Pathologie Animale, Bingerville BP206, Côte d’Ivoire; chymann@hotmail.com

**Keywords:** virus, hepadnavirus, *Orthohepadnavirus*, discovery, hybridization capture, bovid, artiodactyls, sequencing

## Abstract

New technologies enable viral discovery in a diversity of hosts, providing insights into viral evolution. We used one such approach, the virome capture sequencing for vertebrate viruses (VirCapSeq-VERT) platform, on 21 samples originating from six dead Maxwell’s duikers (*Philantomba maxwellii*) from Taï National Park, Côte d’Ivoire. We detected the presence of an orthohepadnavirus in one animal and characterized its 3128 bp genome. The highest viral copy numbers were detected in the spleen, followed by the lung, blood, and liver, with the lowest copy numbers in the kidney and heart; the virus was not detected in the jejunum. Viral copy numbers in the blood were in the range known from humans with active chronic infections leading to liver histolytic damage, suggesting this virus could be pathogenic in duikers, though many orthohepadnaviruses appear to be apathogenic in other hosts, precluding a formal test of this hypothesis. The virus was not detected in 29 other dead duiker samples from the Côte d’Ivoire and Central African Republic, suggesting either a spillover event or a low prevalence in these populations. Phylogenetic analysis placed the virus as a divergent member of the mammalian clade of orthohepadnaviruses, though its relationship to other orthohepadnaviruses remains uncertain. This represents the first orthohepadnavirus described in an artiodactyl. We have tentatively named this new member of the genus *Orthohepadnavirus* (family *Hepadnaviridae*), Taï Forest hepadnavirus. Further studies are needed to determine whether it, or some close relatives, are present in a broader range of artiodactyls, including livestock.

## 1. Introduction

Partially double stranded DNA viruses belonging to the genus *Orthohepadnavirus* infect a number of species of nonhuman primates [1,2,3,4], bats [5,6,7,8], rodents [9,10,11,12], and the domestic cat [13]. The type species of the genus, *Hepatitis B virus* (HBV), remains a major public health concern in humans; in 2015 there were an estimated 257 million people with chronic HBV infections resulting in an estimated 887,000 deaths [14]. In animal host species, infection with orthohepadnaviruses results in signs ranging from hepatitis, to hematomas, and hepatocellular carcinomas (e.g., in woodchucks (*Marmota monax*) [9], arctic ground squirrels (*Spermophylus parryi kennicotti*) [10], and woolly monkey (*Lagothrix lagotricha*) [1]). In vitro studies showed that bat hepatitis viruses have the potential to infect human hepatocytes, highlighting the zoonotic potential of orthohepadnaviruses [15]. Similarly, recombination events between human and primate HBV, and detection of likely transmission events between humans and chimpanzees [16], highlight the potential for cross-species transmission and the importance of understanding orthohepadnavirus ecology and evolution in wildlife [17]. Here, we report the discovery of a novel orthohepadnavirus in a deceased wild Maxwell’s duiker (*Philantomba maxwellii*) in Taï National Park, Côte d’Ivoire. The discovery of this virus in a bovid represents the first indication of hepadnaviruses in artiodactyls, expanding the potential host range of these viruses to a mammalian order that includes many important livestock species.

## 2. Methods and Results

In the course of a long-term research project on wildlife mortality in Taï National Park, Côte d’Ivoire and Dzanga-Sangha Protected Areas (DSPA), Central African Republic, necropsy samples were systematically collected by on site veterinarians and preserved for analyses [18,19,20]. The carcass positive for the novel orthohepadnavirus, was found by field assistants working with the Taï Chimpanzee Project in January 2015 (GPS coordinates: latitude: 5.854, longitude: −7.299). The carcass was found in the core research area of the Taï Chimpanzee Project, where human hunting and trapping activities are rare. We found no evidence on the carcass suggestive of human involvement in the death of this animal, nor evidence of external lesions suggestive of trauma prior to death. The veterinarian on site performed a necropsy on the day after the carcass was found. The liver of this animal was swollen and parts of jejunum and duodenum appeared to be hemorrhagically infiltrated, though maggots were already present on and in the carcass, and advanced autolysis precluded a detailed histopathological description and made an assessment of the degree of swelling and hemorrhage difficult. Samples of heart blood, heart, duodenum, jejunum, kidney, liver, lung, and spleen were collected, fixed in formalin or stored fresh in liquid nitrogen and transported on dry ice to the Robert Koch Institute, Berlin, Germany where they were stored at −80 °C until further analysis was performed. Initially, 21 samples from 6 duikers were analyzed (Table S1) that were negative for other known wildlife pathogens that cause mortality in this ecosystem (i.e., orthopoxviruses [21], *Bacillus cereus* biovar *anthracis* [20]; tested using the diagnostic systems described in [22] and [20], respectively).

Nucleic acids were extracted; frozen tissue samples were using the QIAamp Viral RNA mini Kit (Qiagen, Hilden, Germany) according to the manufacturer’s instructions, but modified the protocol by homogenizing samples with 280 µL AVL buffer and 20–30 ceramic beads in a Fastprep-24 homogenizer (4 m/s for 30 s followed by a cooling step for 2 min on dry ice, for a total of 3 homogenization cycles: MP Biomedicals). By not using RNase, this kit extracts total nucleic acids (TNA). Extractions were also performed without carrier RNA. On the extracted nucleic acid, we performed high throughput sequencing using the virome capture sequencing for vertebrate viruses (VirCapSeq-VERT) platform [23]. For each batch of libraries that were pooled for capture, we also processed salmon sperm DNA as negative control (Table S1). Briefly, TNA was reverse transcribed using SuperScript IV (Thermo Fisher, Waltham, MA, USA) with random hexamers, and the cDNA was RNase H treated prior to second-strand synthesis with Klenow fragment (New England Biolabs, Ipswich, MA, USA). The resulting double-stranded cDNA/DNA mix was sheared by ultrasonication to an average fragment size of 200 bp following manufacturer’s instructions (Covaris E210 focused ultrasonicator). Sheared product was purified using AxyPrep beads (Axygen, Union City, CA, USA) and libraries were prepared with KAPA Hyper Prep kits (KAPA) and custom unique dual barcoding indices. Library quality and quantity was assessed using a Bioanalyzer (Agilent, Santa Clara, CA, USA), before hybridization capture with VirCapSeq-VERT probes [23] and sequencing on an Illumina HiSeq 2500 system. Sequenced reads were demultiplexed with cutadapt (v1.15) and quality filtered and trimming with PRINSEQ software (v 0.20.3). Reads were filtered if the average quality score of a read was less than thirty, if the minimum length was less than 50, and if the entropy was less than 70. The first nine bases and the last base of reads were further trimmed. The full Maxwell’s duiker genome has not been sequenced; therefore, we used a *Bos taurus* genome (GCF_000003055.6) for host subtraction; a Bowtie index was created for the host genome and mapping of the filtered reads was performed using bowtie2 mapper with default settings. Reads that matched the host genome at ≥90% identity were discarded. Host subtracted reads were then assembled using Mira (v 4.0) with SOLEXA settings, with a minimum overlap of 20 between the reads and a minimum of three reads per contig. Contigs and singletons were then collapsed using CD-HIT at 100% identity and subjected to homology search by MegaBlast against the GenBank nucleotide database and sequences not assigned at the nucleotide level were screened by BLASTx to detect divergent or potentially new viruses. Based on the BLAST results, contigs and singletons were assigned at family, genus, species, and GenBank accession number level to identify the most closely related GenBank matches. Hepadnavirus sequences were found in the liver and duodenum of the same animal (Table 1). We detected no other viruses in the liver or duodenum of this animal using VirCapSeq-Vert.

To assemble the full genome of the detected virus, tentatively named Taï Forest hepadnavirus, we downsampled to 1,000,000 reads with SEQTK to make subsequent bioinformatics steps more tractable (https://github.com/lh3/seqtk). Reads were trimmed using Trimmomatic v0.38, removing the leading and trailing reads below Q30, and clipping any part of the read where the average base quality across 4 bp was less than 30 [24]. Nineteen contigs longer than 500 nt were assembled using Spades and default settings [25]. BLAST alignment against the NCBI database indicated that two contigs were hits to orthohepadnavirus sequences. These contigs did not overlap, so we extended them by iteratively mapping our trimmed reads against these contigs with Geneious v11.1.5 [26] using standard settings (5 iterations: http://www.geneious.com). We generated a majority rule consensus sequence for each of those two contigs, requiring a minimum of 10× coverage for a base to be called. This resulted in two contigs, one of 2026 nt that did not contain a poly-A tail, and another, that included a poly-A tail towards the end, with 134 bp following the poly-A tail. We removed the poly-A tail and the 134 nt following it, and ran a *de novo* assembly of the two contigs, resulting in an overlap of 191 nt. We split the combined long contig into two parts and re-aligned them with Geneious using default settings, which resulted in an overlap with 100% identity, suggesting that we had sequenced a 3128 nt circular genomic sequence.

After trimming and quality filtering as above, we mapped the entire read set obtained for the positive duiker sample to this circular genome using BWA [27], resulting in 3,569,327 reads mapping from the positive liver sample and 1330 reads from the duodenum sample (deduplicated = 633,930 and 326 unique reads, respectively; Table S1). This represented 100% genome coverage at a minimum depth of 10× for the liver sample and 10.2% and 38.5% genome coverage at 10× and 1×, respectively, for the duodenum sample. As shown in Table S1, in sample pools with highly positive specimens we encountered background noise, which in batch 2 amounted to 11 to 72 reads (or 0.001–0.007% of the highest signal, when normalized to 10 million total reads generated). This background was not considered as biologically meaningful as it stayed below our positivity cut-off of 0.015% of the highest signal, or not more than 3× the read count detected in the negative salmon sperm control that ran in that pool (Table S1).

**Figure 1:**
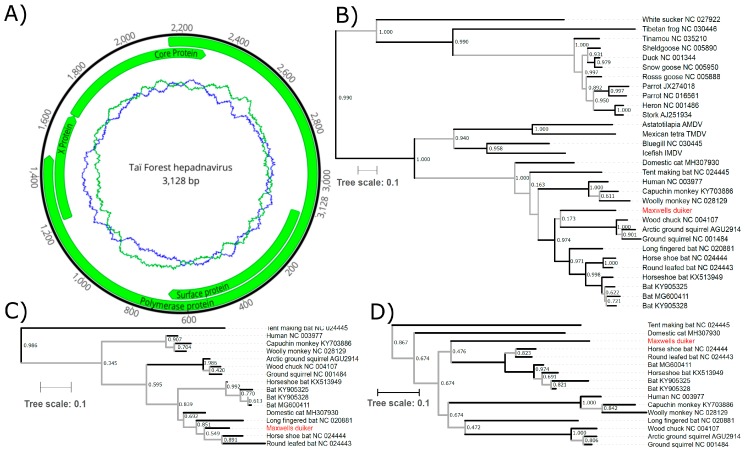
(**A**) The genome organization of Taï Forest hepadnavirus. The innermost circles represent the GC (blue) and AT (green) content along a 50 nt sliding window. (**B**) Phylogenetic relationship of Taï Forest hepadnavirus to other viruses of the family Hepadnaviridae. Maximum likelihood phylogeny constructed using the amino acid sequence of the polymerase ORF from a range of vertebrate hepadnaviruses; and (**C**) using the core and (**D**) the surface protein ORFs in comparison to the mammalian orthohepadnaviruses; the common name of the host is indicated at the branch labels, along with each virus’s accession number. GenBank sequences were aligned to Taï Forest hepadnavirus ORF sequences using MAFFT (v7.307) and we selected conserved blocks using Gblocks, as implemented in SeaView V4 [28]. PhyML with smart model selection [29], and the Bayesian Information Criterion and subtree pruning and regrafting (SPR) was applied as the tree improvement approach, with otherwise default settings for tree building (selected models: polymerase ORF = LG + G + I + F; core ORF = JTT + G; surface ORF = JTT + G + F). We estimated the best-fitting root of these phylogenies using the heuristic residual mean squared function in the program TempEst, which minimizes the variance of root-to-tip distances [30]. To further assess the confidence in our phylogenetic trees, BMCMC analyses were run on each amino acid alignment using BEAST v1.10.4 under the assumption of a relaxed log-normal molecular clock and with tree shape modeled according to a birth-death speciation model and the amino acids substitution model supported by PhyML’s smart model selection [31]. We examined the output of three runs for convergence and appropriate sampling of the posterior using Tracer v1.7.1 [32] before merging runs using LogCombiner v1.10.4 [33]. The best representative tree was then identified from the posterior set of trees and annotated with TreeAnnotator v1.10.4 (distributed with BEAST). Branch support was assessed using Shimodaira-Hasegawa-like approximate likelihood ratio tests (SH-like aLRT), with branches supported by SH-like aLRT values < 0.95 and/or posterior probabilities <0.95 in the Bayesian Markov chain Monte Carlo tree indicated in gray. Branch lengths are representative of substitutions per site. SH-like aLRT values are indicated at each node.

We annotated the circular genome generated from the liver sample in Geneious based on similarity to the long-fingered bat hepatitis *B virus* (NC_020881) and human hepatitis *B virus* (NC_003977) by using a 50% identity threshold to transfer reference annotations (Figure 1A). We also examined the Pfam alignments (https://pfam.xfam.org). Whereas P, X, and PreC/C open reading frame (ORF) sequences were readily recognizable, highly divergent sequence between the preS1 and S ORFs obscure the potential start position of preS2 sequence. Two 11-nt direct repeats located at the start of the preC and the end of the P coding sequence (DR1 and DR2, TTCACCTgTGC) were well conserved when compared to repeats found in the same positions in hepatitis B viruses (TTCACCTcTGC).

To confirm the presence of the virus in these and other duiker samples, we designed four PCR systems in conserved regions of the genome (Table 2) identified through alignment with other mammalian orthohepadnavirus genomes using MAFFT [34]. In addition, we tested the HBVS3s and HBVS3as primers developed by Schaefer, et al. [35]. All PCR systems were tested on the liver sample in a total volume of 25 µL containing 0.2 mM dNTP (with dUTP replacing dTTP), 4 mM MgCl_2_, 0.2 µM of each primer, 1.25U Platinum^®^ Taq Polymerase (Invitrogen, Waltham, MA, USA), 10× PCR Buffer (Invitrogen) and PCR-grade water. Each reaction was seeded with 5 µL TNA and cycling conditions were: 95 °C for 5 min, 35 cycles of [95 °C for 30 s, annealing temp for 30 s (Table 2), 72 °C for 1 min], followed by a final extension at 72 °C for 7 min. All primer sets generated amplification products, which were visualized on agarose gels (data not shown). Amplification products were cleaned using ExoSAP-IT^®^ (Affymetrix, Santa Clara, CA, USA). Sequencing reactions were performed with the primers used for amplification and the BigDye Terminator v3.1 cycle kit (Thermo Fisher, Waltham, MA, USA) and Sanger sequenced in both directions with the ABI PRISM^®^ 3100 Genetic Analyzer (Applied Biosystems, Waltham, MA, USA). Chromatograms were evaluated using Geneious and were 100% matches to the genome generated by VirCapSeq-VERT.

A real-time assay was tested for each primer set, using a reaction volume of 12.5 μL containing 6.25 μL GoTaq^®^ qPCR Master Mix (Promega, Fitchburg, WI, USA), 0.2 μM of each primer and 1 μL TNA extract of the positive duiker liver or 1 μL diluted PCR product. Standards were generated by serial dilution of PCR products. Cycling conditions were 95 °C for 5 min, 40 cycles [95 °C for 15 s, annealing temp (Table S1) for 1 min]. Quantitative PCRs (qPCRs) were run on a BioRad CFX96 measuring fluorescence after each extension phase. Results were analyzed using BioRad Maestro software. Based on these results, the most sensitive of the primer sets (Orthohep_2F/Orthohep_2R) was chosen to measure viral copy numbers in all available tissues from the infected duiker to assess viral organ distribution (Table 3). The observed viral copy numbers in the blood were in the range of that found in humans with an active chronic infection that leads to liver damage [36]. This potentially implicates the virus in the duiker’s death, although further studies are needed to understand the impact of Taï Forest hepadnavirus on duikers, as many hepadnaviruses appear to be apathogenic in other mammalian hosts [11], precluding a formal test of this hypothesis. In this duiker we did not detect other pathogens that can cause duiker mortality in this ecosystem (i.e., orthopoxviruses [21], *Bacillus cereus* biovar *anthracis* [20]; filoviruses [37]), though it is certainly possible that the duiker died of causes unrelated to this Taï Forest hepadnavirus. The highest copy numbers were detected in the spleen and blood, and viral copies were detected in all other tissues tested except for the jejunum (Table 3). Orthohepadnavirus replication outside of the liver in lymphoid cells in the spleen has been proposed in woodchucks [38,39], but understanding the tropism of Tai Forest hepadnavirus in more animals and the determinants of orthohepadnaviruses more generally remain important areas of future research. We also used this qPCR to test samples of 23 additional dead duikers found in the Côte d’Ivoire and the Central African Republic (Table S2; collected in areas where human hunting and trapping is rare and with no indication of anthropogenic causes of mortality), alongside the samples previously assessed by VirCapSeq-VERT. We detected no additional positive samples, confirming the negative results found in the VirCapSeq-VERT experiment.

As with other hepadnaviruses, the reconstruction of phylogenetic relationships is not straightforward given the diverse results obtained for various coding regions and analyses. We examined the P, C and S amino acid sequences to assess the phylogenetic relationship of Taï Forest hepadnavirus to other representative members of the family *Hepadnaviridae*. Maximum likelihood trees were constructed, including viruses from both genera, *Avihepadnavirus* and *Orthohepadnavirus*, as well as unassigned hepadnaviruses were constructed (Figure 1B). For the shorter, more diverse sequences of the C and S ORFs, the analysis was restricted to the mammalian orthohepadnaviruses (Figure 1C,D). To assess potential long-branch effects and whether an outgroup affected tree topology, the phylogeny was also constructed using the P ORF of only the mammalian viruses (Supplementary Figure S1A), and for the C and S ORFs including the bluegill virus as an outgroup (Supplementary Figure S1B,C).

## 3. Discussion

Our data indicate that Taï Forest hepadnavirus is sufficiently different from classified orthohepadnaviruses to represent a separate, likely novel taxon (Figure 1 and Figure S1). Although the precise phylogenetic relationship of the virus to other members of the genus is not clarified, as indicated by the often low statistical support of major branches (Figure 1 and Figure S1), our data add to the growing body of evidence demonstrating a vast diversity of these viruses [1,2,3,4,5,6,7,8,9,10,11,12,13], whose discovery and characterization will provide new insights into the origins and evolution of the hepatitis *B viruses* [40]. The analysis of the largest and most conserved P ORF sequence indicates the Tai Forest hepadnavirus falls within a clade including most known bat and all known rodent orthohepadnaviruses (Figure 1B), though this topology was not supported when the more divergent avihepadnaviruses were excluded from the analysis (Figure S1). Further discovery and sequencing of orthohepadnaviruses from a larger diversity of mammals will likely help to resolve this phylogenetic uncertainty.

In conclusion, we report the first orthohepadnavirus detected in a member of the order Artiodactyla, with considerable viral copy numbers in blood and several organs that are suggestive of widespread viremia. These results provide further evidence for a broad unexplored diversity of orthohepadnaviruses in mammals [41,42]. The low prevalence of the Taï Forest hepadnavirus encountered in the available sample set indicates that a spillover event from another species cannot be ruled out at this point. The scavenging behavior that has been reported for duikers might increase the likelihood for such a cross-species transmission [43,44], but more research on the orthohepadnaviruses circulating in the mammalian community of Taï National Park, Côte d’Ivoire is needed to clarify this. Given our results, understanding the potential impact of orthohepadnaviruses on animal health, including livestock species, represents another important area of future research. Serological and histopathological investigations of carcasses representing a broader diversity of species will allow us to assess the extent of exposure and diversity of orthohepadnaviruses in this ecosystem, as well as potential associations of infection with disease [45]. Further investigations into the evolution of these viruses in a broader range of hosts will provide a clearer understanding of the processes (co-divergence vs. host-switching) that underlie hepadnavirus diversification.

## Figures and Tables

**Table 1:** Read processing of samples containing hits to a orthohepadnavirus.

Tissue	Total Reads	Reads after Filtering	Reads after Host Subtraction	Reads with Top Blast Hit to a Orthohepadnavirus
Liver	10,548,728	10,111,516	9,105,066	2,475,043
Duodenum	9,776,183	9,082,857	6,249,455	964

**Table 2:** Primers designed for sequencing and quantification of Taï Forest hepadnavirus.

Primer Name	Sequence (5’ -> 3’)	Annealing Temperature (°C)
Orthohep_1F	TGGTGGACTTCTCTCAGTTTTCC	56
Orthohep_1R	TGATAAAACGCCGCAGACAC	
Orthohep_1F	TGGTGGACTTCTCTCAGTTTTCC	57
Orthohep_1Rb	AGATGAGGCATAGAACCAGGA	
Orthohep_2F	TGCTTAGCCATCCCTTCGTCA	59
Orthohep_2R	GGCCCCCAGTACCACATCAT	
Orthohep_4F	CACAGGTGAAGCGAAGGACA	58
Orthohep_4R	CCCCAAWACCAVATCATCCATATA	

**Table 3:** Estimated viral copies per duiker genome. To estimate the size of the Maxwell’s duiker genome we calculated the average genome size for bovids in the Animal Genome Size Database, Release 2.0 (www.genomesize.com), using the average species genome size for sheep (*Ovis aries*) and domestic cattle (*Bos taurus*), for which there were multiple entries. A haploid bovid genome was estimated to roughly equal 3.7 pg, so that one Maxwell’s duiker cell would contain approximately 7.4 pg of DNA.

Tissue	Extract Conc. (ng/µL)	Duiker Genomes/µL	Viral Copies/µL	Viral Copies/Duiker Genome
Heart blood	5.9	801.4	4019.4	5.0
Heart	3.9	520.3	831.4	1.6
Liver	7.8	1059.5	3205.0	3.0
Spleen	2.2	290.5	50,203.6	172.8
Lung	1.8	243.2	4903.6	20.2
Kidney	9.1	1232.4	283.8	0.2
Duodenum	17.7	2391.9	939.1	0.4
Jejunum	12.0	1621.6	0.0	0.0

## Supplementary Materials

The following are available online at https://www.mdpi.com/1999-4915/11/3/279/s1, Figure S1: Phylogenetic relationship of Taï Forest hepadnavirus to other viruses of the family *Hepadnaviridae*, Table S1: Reads mapping to Taï Forest hepadnavirus following enrichment by VirCapSeq-VERT, Table S2: List of duiker tissues collected during necropsies and tested for the Taï Forest hepadnavirus.; Files related to the phylogenetic analysis are available via: https://github.com/jangogarten/Maxwells-duiker-hepatitis-B-virus. All reads from the two positive samples are avaialbe under SRA accession number: PRJNA526079 and the full genome has been uploaded to GenBank with the Accession Number: MK620908.
