# Supplementary material for: A Novel Orthohepadnavirus Identified in a Dead Maxwell’s Duiker (Philantomba maxwellii) in Taï National Park, Côte d’Ivoire
Source: Viruses. 2019 Mar 19;11(3):279. doi: 10.3390/v11030279 (PMC6466360; doi:10.3390/v11030279)
Supplement: Supplementary file 1 [file viruses-11-00279-s001.pdf]

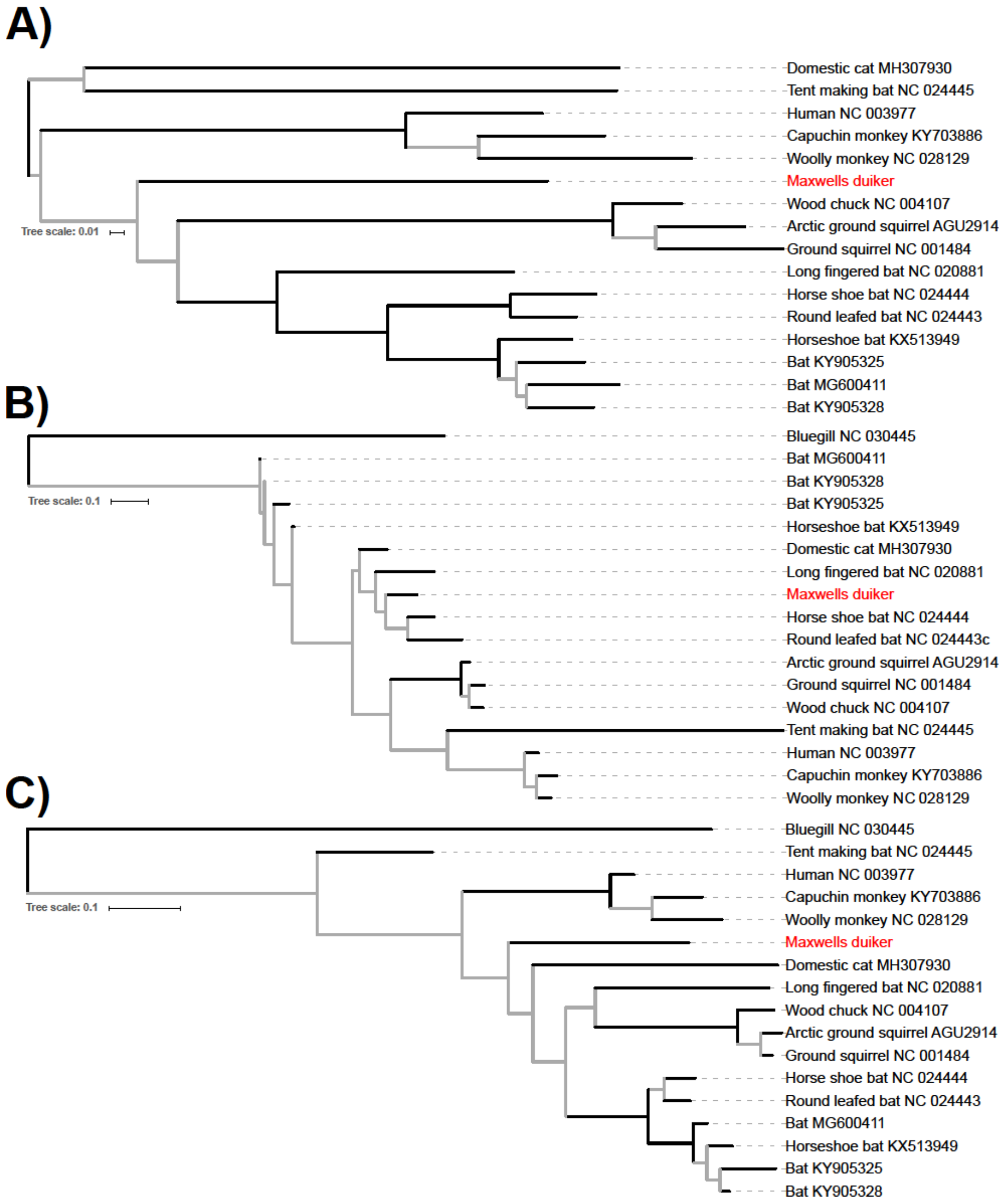


**Figure 1S.** Phylogenetic relationship of Taï Forest hepadnavirus to other viruses of the family *Hepadnaviridae*. **A)** Maximum likelihood phylogeny constructed using the amino acid sequence of the polymerase ORF from mammalian orthohepadnaviruses; and **B)** the core and **C)** the surface protein ORFs from mammalian orthohepadnaviruses with bluegill hepatitis B virus as an outgroup. The common name of the host indicated at the branch labels, along with each virus's accession number. Sequence processing and phylogenetic analyses were performed as outlined in Figure 1 (selected models: polymerase ORF=LG+G+I+F; core ORF: JTT+G; surface ORF: AB+G+I+F). Branch support was assessed using Shimodaira-Hasegawa-like approximate likelihood ratio tests (SH-like aLRT), with branches supported by SH-like aLRT values < 0.95 and/or posterior probabilities <0.95 in the Bayesian Markov chain Monte Carlo tree indicated in gray. Branch lengths are representative of substitutions per site.

**Table S1.** Reads mapping to Taï Forest hepadnavirus following enrichment by VirCapSeq-VERT.

| Animal ID | Tissue | Batch | Total reads [N] | Reads after quality filtering [N] | De-duplicated reads mapping to Taï Forest hepadnavirus [N] | 1× genome coverage [%] | 10× genome coverage [%] | 100x genome coverage [%] |
|---|---|---|---|---|---|---|---|---|
| Control | salmon sperm | 1 | 13,004,643 | 11,477,668 | 0 | 0.0 | 0.0 | 0.0 |
| 2106 | blood | 1 | 9,293,627 | 7,093,405 | 0 | 0.0 | 0.0 | 0.0 |
| 2106 | thymus | 1 | 6,256,447 | 4,635,529 | 0 | 0.0 | 0.0 | 0.0 |
| 2141 | abdominal liquid | 1 | 9,001,096 | 6,923,635 | 0 | 0.0 | 0.0 | 0.0 |
| Control | salmon sperm | 2 | 13,283,014 | 10,496,492 | 34 | 13.9 | 3.5 | 0.0 |
| 516 | liver | 2 | 8,431,930 | 6,913,116 | 35 | 21.5 | 3.3 | 0.0 |
| 522 | liver | 2 | 13,440,618 | 11,018,108 | 19 | 13.5 | 0.8 | 0.0 |
| 533 | liver | 2 | 11,470,268 | 9,348,492 | 39 | 19.4 | 2.1 | 0.0 |
| 2054 | liver | 2 | 10,548,728 | 8,712,701 | 633,930 | 100.0 | 100.0 | 90.9 |
| 2106 | liver | 2 | 13,971,570 | 11,386,699 | 11 | 6.1 | 0.0 | 0.0 |
| 2141 | liver | 2 | 16,329,153 | 13,425,803 | 72 | 23.0 | 4.8 | 0.0 |
| Control | salmon sperm | 3 | 7,701,849 | 6,044,670 | 0 | 0.0 | 0.0 | 0.0 |
| Control | salmon sperm | 4 | 13,083,897 | 11,579,184 | 0 | 0.0 | 0.0 | 0.0 |
| 516 | lung | 4 | 6,874,886 | 5,840,900 | 0 | 0.0 | 0.0 | 0.0 |
| 522 | lung | 4 | 12,628,478 | 11,532,983 | 0 | 0.0 | 0.0 | 0.0 |
| 533 | lung | 4 | 11,759,758 | 10,686,006 | 0 | 0.0 | 0.0 | 0.0 |
| 2106 | lung | 4 | 10,884,163 | 9,678,536 | 0 | 0.0 | 0.0 | 0.0 |
| 2141 | lung | 4 | 8,199,692 | 7,217,568 | 0 | 0.0 | 0.0 | 0.0 |
| Control | salmon sperm | 6 | 8,318,734 | 6,762,513 | 0 | 0.0 | 0.0 | 0.0 |
| 522 | mesenteric lymph node | 6 | 9,791,083 | 8,380,341 | 0 | 0.0 | 0.0 | 0.0 |
| 533 | lymph node | 6 | 12,853,198 | 10,784,007 | 0 | 0.0 | 0.0 | 0.0 |
| 2106 | mesenteric lymph node | 6 | 5,500,016 | 4,507,359 | 0 | 0.0 | 0.0 | 0.0 |
| Control | salmon sperm | 7 | 17,839,711 | 13,871,470 | 0 | 0.0 | 0.0 | 0.0 |
| 522 | kidney | 7 | 5,252,896 | 3,931,313 | 0 | 0.0 | 0.0 | 0.0 |
| 2106 | kidney | 7 | 7,737,228 | 5,704,512 | 0 | 0.0 | 0.0 | 0.0 |
| 2141 | kidney | 7 | 7,619,632 | 6,019,141 | 0 | 0.0 | 0.0 | 0.0 |
| Control | salmon sperm | 8 | 8,915,925 | 7,902,488 | 0 | 0.0 | 0.0 | 0.0 |
| 2054 | duodenum | 8 | 9,776,183 | 8,738,188 | 326 | 38.5 | 10.2 | 1.6 |

**Table S2.** List of duiker tissues collected during necropsies and tested for the Taï Forest hepadnavirus.

| Sample ID | Species | Tissues | Country | Cause of death |
|---|---|---|---|---|
| 1753 | unknown Cephalophini | Liver | CAR | |
| 1754 | unknown Cephalophini | Liver | CAR | *B*CBV*A* |
| 2184 | *Cephalophus monticola* | Liver | CI | *B*CBV*A* |
| 516 | *Philantomba maxwellii* | Lung, liver | CI | |
| 522 | *Philantomba maxwellii* | Kidney, liver, lung, lymph node | CI | |
| 533 | *Philantomba maxwellii* | Lung, liver, lymph node | CI | |
| 534 | *Philantomba maxwellii* | Liver | CI | |
| 1888 | *Philantomba maxwellii* | Liver | CI | *B*CBV*A* |
| 2054 | *Philantomba maxwellii* | Duodenum, heart, heart blood, jejunum, kidney, liver, lung, spleen | CI | |
| 2055 | *Philantomba maxwellii* | Liver | CI | *B*CBV*A* |
| 2106 | *Philantomba maxwellii* | Blood, liver, lymph node | CI | |
| 2141 | *Philantomba maxwellii* | Abdominal liquid, kidney, liver, lung | CI | |
| 509 | unknown Cephalophini | Liver | CI | *B*CBV*A* |
| 842 | unknown Cephalophini | Liver | CI | *B*CBV*A* |
| 843 | unknown Cephalophini | Liver | CI | *B*CBV*A* |
| 1860 | unknown Cephalophini | Liver | CI | *B*CBV*A* |
| 1861 | unknown Cephalophini | Liver | CI | *B*CBV*A* |
| 1870 | unknown Cephalophini | Liver | CI | *B*CBV*A* |
| 1872 | unknown Cephalophini | Liver | CI | *B*CBV*A* |
| 1873 | unknown Cephalophini | Liver | CI | *B*CBV*A* |
| 1890 | unknown Cephalophini | Liver | CI | *B*CBV*A* |
| 1901 | unknown Cephalophini | Liver | CI | *B*CBV*A* |
| 2049 | unknown Cephalophini | Liver | CI | *B*CBV*A* |
| 2051 | unknown Cephalophini | Liver | CI | *B*CBV*A* |
| 2052 | unknown Cephalophini | Liver | CI | *B*CBV*A* |
| 2059 | unknown Cephalophini | Liver | CI | *B*CBV*A* |

| | | | | |
|---|---|---|---|---|
| 2070 | unknown Cephalophini | Liver | CI | *BC*BV*A* |
| 2185 | unknown Cephalophini | Liver | CI | *BC*BV*A* |
| 2193 | unknown Cephalophini | Liver | CI | *BC*BV*A* |
| 65 | unknown Cephalophini | Liver | CI | *BC*BV*A* |

CAR = Central African Republic, CI = Côte d'Ivoire, *BC*BV*A* = *Bacillus cereus* biovar *anthracis*
